# Editorial: Novel Therapeutic Potential for Pituitary Adenylate Cyclase-Activating Polypeptide (PACAP), Vasoactive Intestinal Peptide (VIP) and Related Peptides in Cognition Deficits

**DOI:** 10.3389/fncel.2021.748970

**Published:** 2021-09-13

**Authors:** Lucia Ciranna, Dora Reglodi, Billy K. Chow, David Vaudry

**Affiliations:** ^1^Department of Biomedical and Biotechnological Sciences, University of Catania, Catania, Italy; ^2^Department of Anatomy, PTE-MTA PACAP Research Team, University of Pecs Medical School, Pecs, Hungary; ^3^The University of Hong Kong Pokfulam, Hong Kong, SAR China; ^4^Normandie Univ, UNIROUEN, Inserm, Laboratory of Neuronal and Neuroendocrine Communication and Differentiation, Neuropeptides, Neuronal Death and Cell Plasticity Team, Rouen, France

**Keywords:** PACAP, VIP, synaptic plasticity, learning, cognitive deficit

It is well-known that Pituitary Adenylate Cyclase-Activating Polypeptide (PACAP), Vasoactive Intestinal Peptide (VIP) and related peptides act as neurotrophic and neuroprotective factors in the central nervous system (CNS). The present Research Topic highlights that these peptides also regulate learning and memory. In particular, several of the manuscripts (Ciranna and Costa; Cunha-Reis and Caulino-Rocha; Gilmartin and Ferrara; Johnson et al.; Solés-Tarrés et al.) demonstrate that PACAP modulates synaptic transmission and plasticity in the hippocampus, a brain area crucially involved in learning. It appears that PACAP released by projections from hippocampal hilar mossy cells increases the excitability of dentate gyrus (DG) neurons and modifies contextually-mediated fear memory (Johnson et al.). The circuit involving DG neurons is responsible for fear-related discrimination between similar contexts (Liu et al., 2012), thus a malfunction may disrupt the ability to discriminate between fearful and harmless situations linked to a similar context, a typical symptom of stress-related pathologies such as post-traumatic stress disorder (PTSD). Johnson et al. demonstrates that PACAP plays a crucial role in this process, with important translational implications for PTSD therapy. Interestingly, Gilmartin and Ferrara underlines sex-related differences, with defects in PACAP expression particularly affecting females, which might account for the increased vulnerability of females to PTSD symptoms. The authors also raise open questions concerning the role of PACAP in fear-related learning. As an intriguing speculative hypothesis, PACAP released during stressful events, by enhancing neuronal excitability, might determine which neurons are recruited into a memory circuit.

Cunha-Reis and Caulino-Rocha reviews VIP effects on cognition, illustrating in details the complex hippocampal circuits involving VIP-mediated transmission. This highlights that endogenous VIP is released by a subtype of hippocampal interneurons controlling pyramidal neurons both directly and indirectly through GABAergic interneurons. It appears from their analysis, that a malfunction of VIP-mediated signaling would be responsible for altered excitability of pyramidal neurons, leading to epilepsy. Therefore, PACAP/VIP receptors are promising targets to prevent hippocampal epileptogenesis and subsequent cognitive decline.

New data are emerging regarding endogenous molecules mediating PACAP/VIP effects in the CNS. In particular, PACAP and VIP stimulate the synthesis of Activity-Dependent Neuroprotective Protein (ADNP), an astroglial-secreted protein playing a crucial role in CNS development (Zusev and Gozes, 2004). Sragovich et al. shows that ADNP regulates cognition since ADNP-deficient mice displayed impaired memory in the Morris Water Maze task, which was rescued by intranasal administration of SKIP, a synthetic peptide derived from ADNP. This finding suggests that cognition might be improved by stimulating ADNP production through the PACAP and VIP system.

Several studies demonstrate that PACAP and VIP are involved in pathologies with cognition impairment, such as Alzheimer's disease (AD), Parkinson's disease (PD) and Huntington's disease (HD) (Ciranna and Costa; Solés-Tarrés et al.). Using a transgenic mouse model of AD, Perényi et al. shows cellular damages correlated with deficits of the PACAPergic system on peripheral organs. For instance, Aβ accumulated in kidneys, with a parallel decrease in the expression level of PACAP receptors and of PACAP-activated signaling messengers PKA and CREB. In the same AD model, elevated physical activity protected the kidneys from Aβ accumulation and rescued PACAP receptor expression as well as PACAP-mediated signaling. These results indicate that the neuroprotective action of physical activity in AD might be, at least partly, mediated by PACAP.

Concerning PD, Mosley et al. shows that VPAC2 receptors exert neuroprotection acting through regulatory T cells (Tregs), a subtype of T cells exerting inhibitory control on the immune system. Systemic administration of LBT-3627, a novel selective VPAC2 receptor agonist, increased Treg activity; interestingly, LBT-3627 also reduced brain inflammatory microglia and increased survival of substantia nigra dopaminergic neurons in rat models of PD. Thus, activation of peripheral VPAC2 receptors, by improving Treg function, induced neuroprotection of central dopaminergic neurons. This interesting result opens new challenges to identify the biological cascade underlying peripheral-central crosstalk.

Solés-Tarrés et al. accurately describes the rescue processes of PACAP and VIP in AD, PD, and HD, and decipher the underlying molecular mechanisms, showing the role of each PACAP/VIP receptor subtype. Of note, in AD and HD murine models and PD cellular models, beneficial effects of PACAP are mediated at least in part by stimulation of BDNF production (Rat et al., 2011; Brown et al., 2013; Cabezas-Llobet et al., 2018).

PACAP and VIP deficiency may also contribute to amyotrophic lateral sclerosis (ALS) and multiple sclerosis (MS) non-motor symptomatology. Administration of PACAP and VIP as a possible therapy of ALS and MS is under investigation with respect to neuroprotective effects; future studies would be important to explore the capacity of PACAP and VIP to fight cognitive decline in ALS and MS (Solés-Tarrés et al.).

Ciranna and Costa suggests that Fragile X Syndrome (FXS) patients might benefit from a treatment based on PACAP receptor activation. FXS is a genetic cause of intellectual disability associated with autism, epilepsy, and mood disorders; no specific cure is presently available. Studies from FXS animal models revealed malfunctions in many intracellular signaling pathways, among which a reduction in cyclic AMP (cAMP) levels and downstream pathways (Kelley et al., 2008). PACAP, a very potent stimulator of adenylate cyclase, rescued hippocampal synaptic plasticity in a FXS mouse model (Costa et al., 2018). Therefore, Ciranna and Costa discusses evidence of downregulation of cAMP signaling in FXS patients and FXS animal models, underlining a novel potential of PACAP for FXS therapy.

To come to the point, articles from the present Topic issue highlight that PACAP, VIP, and related peptides play a role in memory and cognition, thus are very promising candidates for novel therapies of cognitive impairment. In support of future clinical studies, this issue also reviews the most suitable administration routes for PACAP, VIP, and related peptides, as well as novel subtype-selective PACAP/VIP receptor agonists with improved systemic stability (Mosley et al.; Solés-Tarrés et al.; Sragovich et al.). Intranasal administration route of exogenous PACAP and VIP in association with excipients (cyclodextrins) improves bioavailability and favors preferential distribution in specific brain areas, increasing the efficiency of the neuropeptide for CNS treatments while limiting its side effects. Taken together, we believe that the reports from this Research Topic will promote development of clinical research based on PACAP, VIP, and related peptides as pharmacological tools for treatment of cognitive deficits.

## Author Contributions

LC wrote the first draft of the manuscript. DR, BC, and DV wrote sections of the manuscript. All authors contributed to manuscript revision, read, and approved the submitted version.

## Conflict of Interest

The authors declare that the research was conducted in the absence of any commercial or financial relationships that could be construed as a potential conflict of interest.

## Publisher's Note

All claims expressed in this article are solely those of the authors and do not necessarily represent those of their affiliated organizations, or those of the publisher, the editors and the reviewers. Any product that may be evaluated in this article, or claim that may be made by its manufacturer, is not guaranteed or endorsed by the publisher.
